# 451. Is Antibody to Nucleocapsid More Prevalent in Individuals with Severe COVID-19?

**DOI:** 10.1093/ofid/ofab466.650

**Published:** 2021-12-04

**Authors:** Hemang Patel, Ashish Bhargava, Daniel Lebovic, Tiziano Scarabelli, Meredith M Coyle, Natali Salaytah, Louis Saravolatz

**Affiliations:** 1 Ascension St John Hospital, Detroit, Michigan; 2 Ascension St John, Grosse Pointe Woods, MI; 3 St. John’s Ascension, Detroit, MI; 4 St John Hospital, Detroit, Michigan

## Abstract

**Background:**

Virus-specific antibodies help to understand the prevalence of infections and the course of the immune response. Humans produce antibodies against the spike and nucleocapsid proteins of SARS-COV-2 virus. Patients with COVID-19 who recover from the infections have higher levels of antibodies to spike proteins. Our study aimed to find the levels of antibodies to spike and nucleocapsid proteins in severe COVID-19.

**Methods:**

A single center prospective study was done at Ascension St John Hospital, Detroit, MI. We included COVID-19 cases diagnosed by reverse-transcriptase polymerase-chain-reaction (RT-PCR). Quantitative measurements of plasma or serum antibodies to nucleocapsid and spike proteins were done in hospitalized patients with acute COVID-19. Using the electronic medical record, we collected data on demographic and clinical information.

**Results:**

A total 24 patients were studied. Of which, 15 patients were suffering from severe and critical COVID 19 and 9 patients were suffering from mild to moderate COVID 19. The mean age (standard deviation) of our cohort was 69 ± 10 years and 60% were males. Common comorbid conditions were hypertension, obesity, and type 2 diabetes. We also noted that severe to critical COVID 19 expressed higher level of antibody to nucleocapsid.

**Conclusion:**

These results display the seroconversion in COVID 19 patients. Our study shows antibody level remain high in severe COVID 19 patients but those are against nucleocapsid protein instead of spike protein.

**Disclosures:**

**All Authors**: No reported disclosures

